# Radio‐Frequency Front‐End‐Free Hybrid Code‐Division and Space‐Division Wireless Multiplexing Using Space‐Time‐Coding Metasurface

**DOI:** 10.1002/nap2.70277

**Published:** 2026-08-31

**Authors:** Yuzhen Chen, Xiaoyi Wang, Guo‐Min Yang, Ya‐Qiu Jin

**Affiliations:** ^1^ Key Laboratory for Information Science of Electromagnetic Waves (MoE) Fudan University Shanghai China; ^2^ College of Electronic and Information Engineering Tongji University Shanghai China

## Abstract

Code‐division multiplexing (CDM) is essential for supporting massive connectivity in wireless networks, but its implementation typically requires complex and costly radio‐frequency (RF) chains. This work presents a simplified wireless CDM system based on a space‐time‐coding metasurface (STCM). By modulating distinct spatial partitions of the metasurface with data streams prespread via orthogonal pseudo‐random codes, the STCM functions as a distributed parallel modulator. This architecture enables the metasurface to perform simultaneous upconversion while directing modulated reflections from multiple spatial partitions toward either shared or distinct directions. Although these data‐carrying waves may physically overlap in the far‐field, the inherent orthogonality of the spread codes ensures that the receiver can successfully decorrelate and recover each multiplexed stream. Experimental validation demonstrates three core scenarios: multistream to a single direction, distinct single‐stream to distinct directions, and multistream to multiple directions (each direction gets multiple streams), all achieved without conventional RF chains. The proposed STCM‐based hybrid CDM and space‐division multiplexing (SDM) architecture enables simultaneous multiuser communication with a radically simplified RF front‐end, paving the way for simplified, high‐capacity wireless systems for future wireless networks.

## Graphical abstract

This work constructs a radio‐frequency front‐end‐free hybrid code‐division and space‐division‐multiplexing wireless communication system built on programmable space‐time‐coding metasurfaces. Orthogonal pseudo‐random codes reliably separate overlapped multistream signals that share identical frequency, time, and spatial resources. Three flexible multiuser directional transmission scenarios are fully validated via comprehensive hardware experiments in a microwave anechoic chamber.

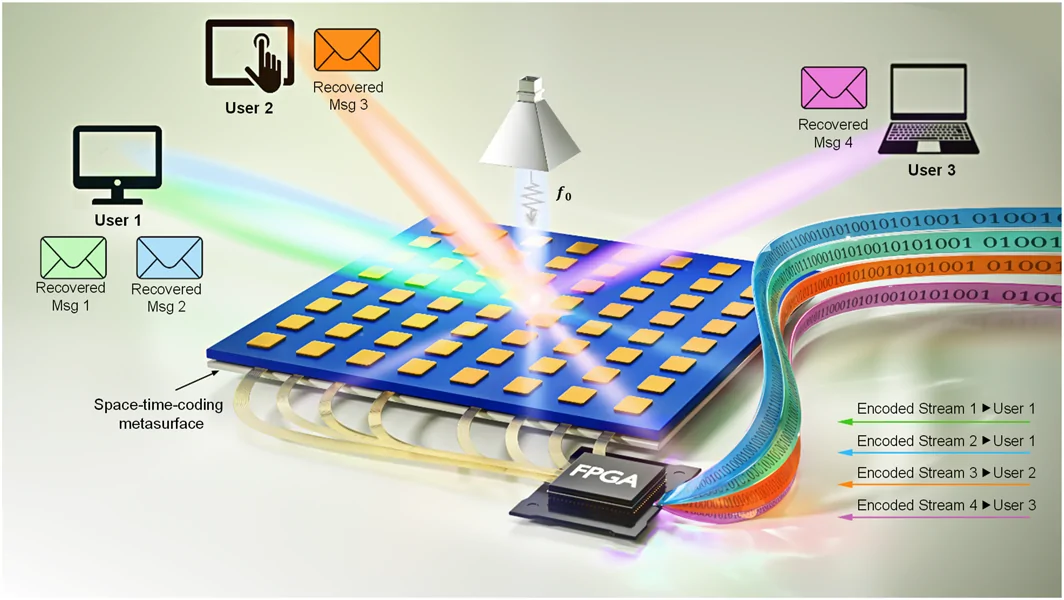

## 1 Introduction

The rise of the Internet of Things (IoT) and the advent of the sixth‐generation (6G) networks are intensifying the demand for wireless systems that support massive connectivity and high spectral efficiency [1, 2]. A fundamental challenge in meeting this demand lies in scaling the user capacity of wireless networks to accommodate an ever‐increasing number of connected devices. Multiplexing technology—which enables multiple users to share limited spectral resources simultaneously—offers a natural and powerful solution to this challenge.

Code‐division multiplexing (CDM), for instance, is a pivotal technique that allows multiple users to share the same frequency band simultaneously, thereby boosting network capacity and resilience [3, 4]. However, conventional CDM depends on intricate radio‐frequency (RF) chains—comprising mixers, filters, amplifiers, and antennas—to generate and process wideband spread‐spectrum signals. This hardware complexity translates into high power consumption, physical bulk, and elevated cost. These drawbacks become particularly prohibitive at higher frequencies, such as the millimeter‐wave bands envisioned for 6G, where the high expense and energy consumption of traditional phased arrays pose major impediments [5]. Simplifying the RF front‐end architecture is therefore a critical step toward scalable multiuser wireless systems.

Metasurfaces are artificially engineered thin‐film structures with tailored electromagnetic properties, which offer an inherently low‐profile and passive platform for wave manipulation [6, 7, 8]. Their exceptional ability to control electromagnetic waves in real time has positioned them as a foundational technology for next‐generation wireless systems [9, 10, 11, 12, 13], with demonstrated applications ranging from dynamic coverage enhancement to holographic multiple‐input multiple‐output (MIMO) systems [14, 15, 16, 17, 18, 19, 20, 21, 22]. Building upon this platform, the recent advancement of space‐time‐coding metasurfaces (STCMs) has introduced temporal modulation as an additional degree of freedom, unlocking dynamic wave control that is directly relevant to wireless communications [23, 24, 25, 26, 27]. STCMs achieve this with a dramatically simplified architecture, where a passive reflective aperture illuminated by a single‐tone source performs modulation and beamforming directly in free space, eliminating the need for per‐channel upconversion chains and active components such as mixers and power amplifiers [28, 29, 30, 31]. This architectural simplicity directly addresses the scalability bottleneck of conventional wireless systems.

In existing STCM‐based communication systems, this simplified architecture has been exploited to implement various multiplexing schemes, including polarization‐division multiplexing (PDM), frequency‐division multiplexing (FDM), and space‐division multiplexing (SDM) [32, 33, 34, 35, 36, 37, 38], as summarized in Table 1. However, regardless of the multiplexing dimension used, all existing schemes rely on narrowband modulation formats and offer no processing gain against interference. Spread‐spectrum modulation provides an effective solution to this limitation [39, 40, 41, 42, 43]. By multiplying each data symbol with a high‐rate pseudo‐random code, the signal bandwidth is greatly expanded, and at the receiver, correlation‐based despreading compresses the desired signal while spreading any narrowband interference across the entire bandwidth, yielding a processing gain that enhances the signal‐to‐interference ratio. When orthogonal pseudo‐random sequences are assigned to different users, this technique enables CDM [44, 45]. Unlike FDM or SDM, which divide resources exclusively among users, CDM allows multiple users to share the same time, frequency, and spatial resources simultaneously through orthogonal codes, achieving more efficient spectrum utilization while providing inherent resilience against narrowband interference and multipath fading. Nevertheless, despite these advantages, CDM has never been introduced into the STCM framework, which presents a clear opportunity.

**TABLE 1:** Comparison between previous research and this work.

Ref.	Channel Number	Multiplexing scheme	Modulation Method	Maximum modulation Frequency	Maximum Data Rate	Tunable Devices
[32]	2	SDM	QPSK	2 MHz	8 Mbps	768 PINs
[33]	3	FDM & SDM	PSK, QAM	10 kHz	60 kbps	41 PINs
[34]	8	FDM & SDM	QPSK	0.8 MHz	50 kbps	512 Varactors
[35]	2	PDM	16‐QAM	2.5 MHz	20 Mbps	288 Varactors
[36]	2	FDM & SDM	OOK	80 MHz	10 Mbps	256 PINs
[37]	1	—	BFSK	312.5 kHz	78.125 kbps	256 PINs
[38]	4	PDM & FDM &SDM	PSK	0.5 MHz	2 Mbps	288 Varactors
This work	4	CDM & SDM	PSK	5 MHz	5 Mbps	300 PINs

Abbreviations: BFSK, Binary Frequency‐Shift Keying; OOK, On–Off Keying; PSK, Phase‐Shift Keying; QAM, Quadrature Amplitude Modulation; QPSK, Quadrature Phase‐Shift Keying.

This paper proposes and experimentally demonstrates a CDM and SDM wireless communication scheme based on STCM, achieving RF‐front‐end‐free multistream transmission over a shared channel through the combination of code‐division and space‐division multiplexing. In the proposed approach, each data stream is first spread by a distinct orthogonal pseudo‐random code and then assigned to modulate a specific spatial partition of the metasurface. When illuminated by a monochromatic carrier wave, the STCM simultaneously upconverts and directs each encoded stream toward designated spatial coordinates. At the receiver, the desired data stream is recovered via correlation with the corresponding orthogonal code, decoupling overlapping transmissions even when multiple streams arrive from the same direction. We investigate three experimental scenarios to validate the scheme: multistream transmission to a single receiver; single‐stream delivery to multiple receivers at distinct angles; and simultaneous multistream transmission to multiple receivers at distinct angles, achieving joint CDM‐SDM operation. In contrast to conventional CDM systems, whose front‐end complexity scales with the number of users, our metasurface‐based scheme performs spreading and beamforming directly in free space via a single passive aperture, keeping the RF front‐end fixed regardless of how many streams are multiplexed. By introducing CDM into the STCM framework, this work adds a new dimension, the code domain, to STCM‐based multiplexing, enhancing both user capacity and interference resilience for future scalable wireless applications.

## 2 Result

### 2.1 Concept and Operating Principles

Figure 1 conceptually illustrates the proposed wireless communication scheme based on an STCM for hybrid CDM and SDM. The metasurface is composed of a two‐dimensional array of programmable unit cells, each integrated with three PIN diodes to enable dynamic control of the reflection phase. In operation, multiple streams of digital data sharing the same spectrum are encoded using a set of binary orthogonal codes to spread their spectra. This matrix ultimately determines the switching states of the PIN diodes integrated on each metasurface unit cell and is accordingly translated into the output logic levels of the corresponding FPGA pins. As a result, when the metasurface is illuminated by a monochromatic incident wave, independent directional transmission of the encoded streams is achieved, allowing users at specific locations to receive the multiple streams directed toward them. The prior knowledge of the orthogonal spread‐spectrum codes enables each user to separate their intended stream from the shared channel and spectrum.

**FIGURE 1:**
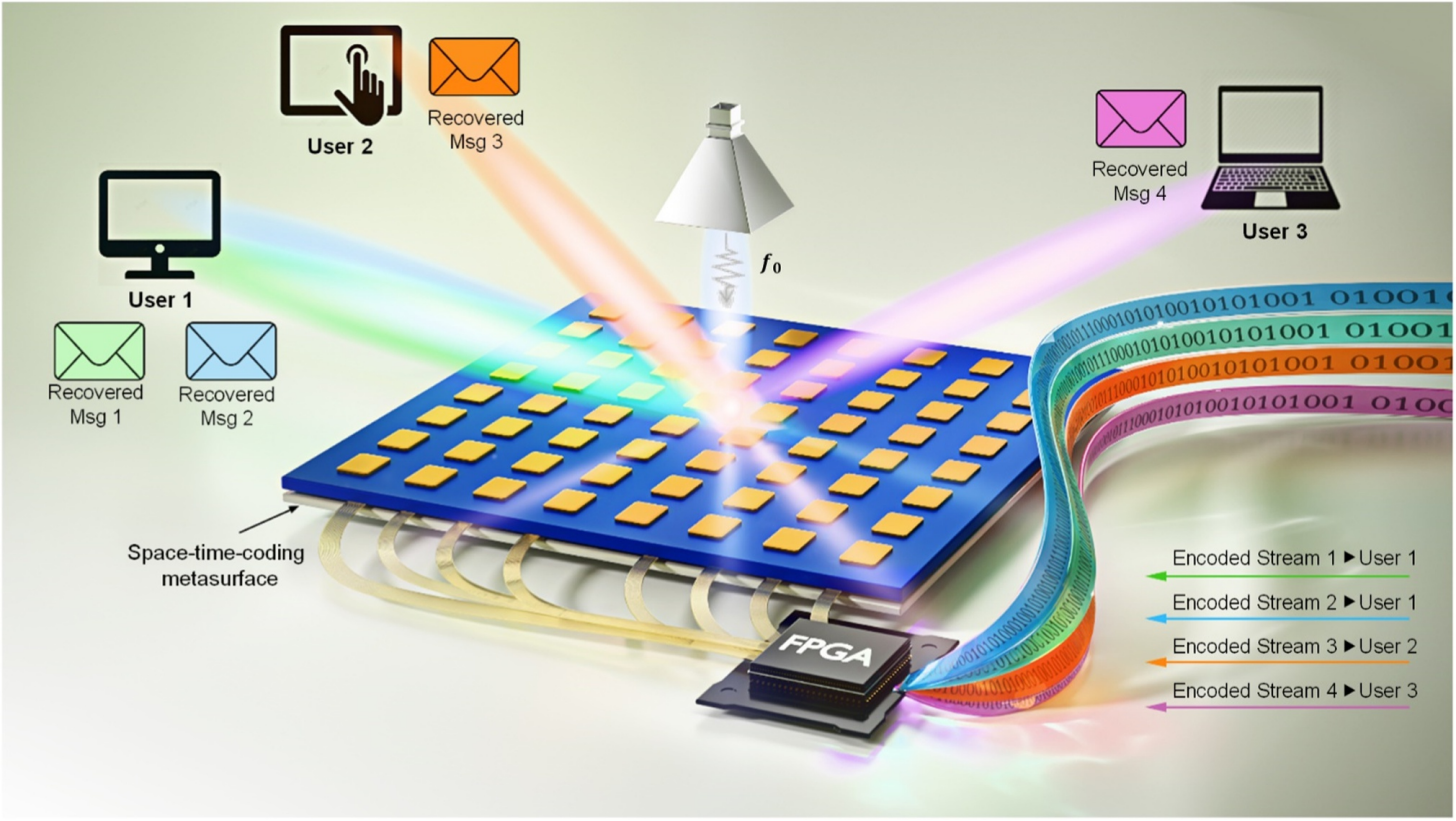
Conceptual illustration of the proposed hybrid CDM and SDM wireless communication scheme using an STCM. The coding matrix ultimately determines the switching states of the PIN diodes integrated on each metasurface unit cell and is accordingly translated into the output logic levels of the corresponding field‐programmable gate array (FPGA) pins. The FPGA outputs corresponding bias voltages to precisely switch the diodes, thereby encoding the phase response of the metasurface. The STCM is partitioned into regions, each assigned a data stream and designed with a phase gradient. Illuminated by a monochromatic wave at *f*_0_, each metasurface region reflects a beam that is simultaneously encoded with its data stream and directed by its phase gradient, realizing parallel multistream transmission. By applying orthogonal code correlation‐based algorithms, receiver antenna 1 despreads both message #1 and message #2 from the composite received signal. Similarly, receiver antenna 2 simultaneously recovers message #3 and message #4. This ability to support both directional multiuser communication and parallel multistream delivery underscores the scheme's flexible reception performance across various communication scenarios.

To better illustrate the operating principle of the proposed STCM‐based wireless CDM, Figure 2 presents a schematic depicting the scenario with two messages to be sent. Figure 2a details the transmitter architecture. The messages to be sent are first transformed into binary bit streams $s_{1}\left(t\right)$ and $s_{2}\left(t\right)$. A pair of 8‐bit Walsh codes $c_{1}\left(t\right)$ and $c_{2}\left(t\right)$ serve as orthogonal codes for spectrum‐spreading because of their strict mathematical orthogonality. To facilitate transmission and processing, all the above binary bit streams are mapped to bipolar symbols, where binary “1” is mapped to “+1” and binary “0” to “−1.” The encoded stream $m_{n}\left(t\right)$ after spreading process for the *n*th data stream can be written as

(1) $$\begin{array}{c} {m_{n}\left(t\right)=s}_{n}\left(t\right)c_{n}\left(t\right), \end{array}$$

**FIGURE 2:**
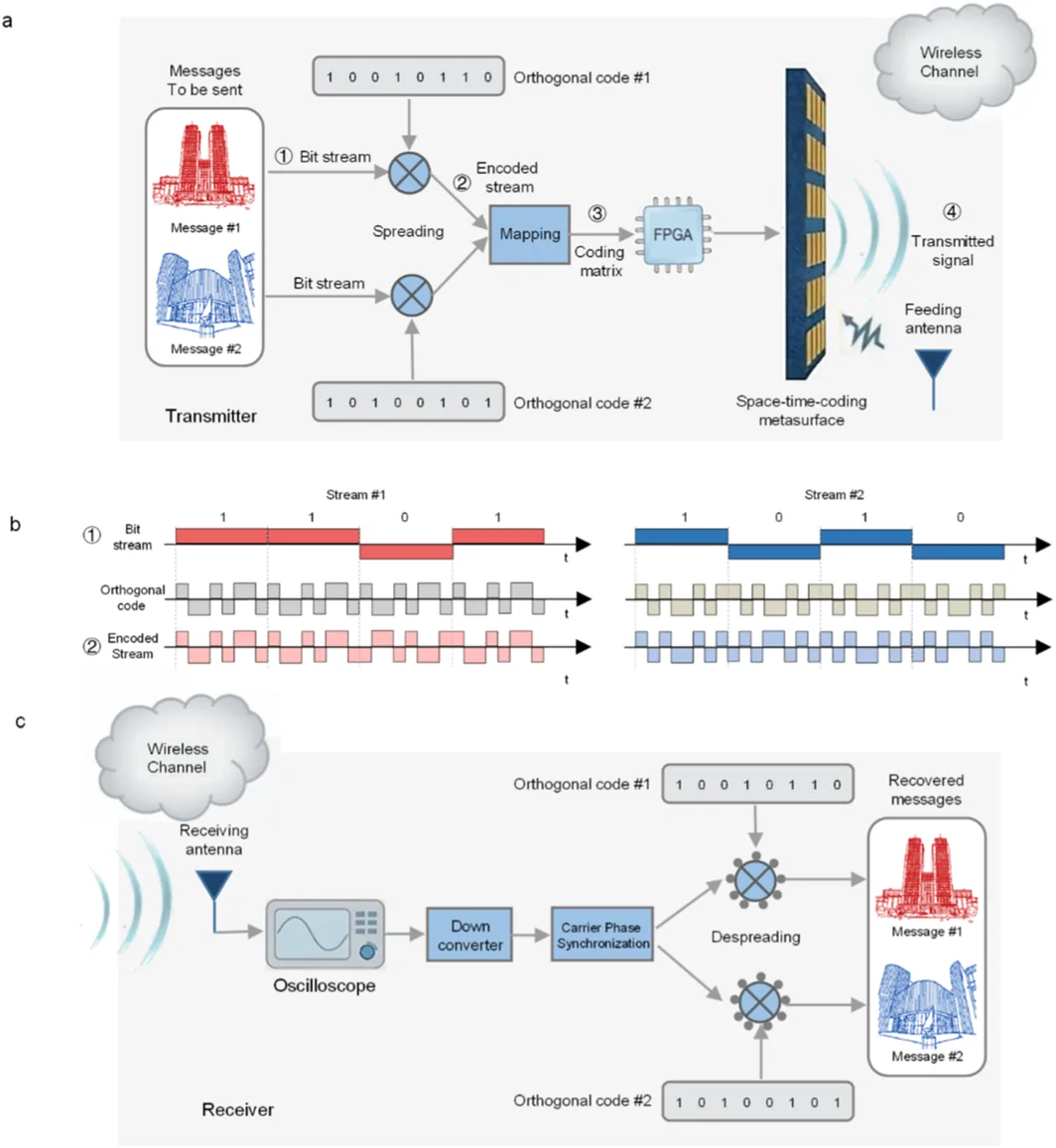
Schematic representation of the dual‐stream wireless communication system based on the STCM. (a) Block diagram of the transmitting process. The messages to be sent are converted into bit streams and then spread using orthogonal codes. The encoded streams with gradient phases are then mapped to the coding matrix for the metasurface, which is prestored in an FPGA. Based on the mapped coding matrix, the FPGA precisely controls the reflection phase of each unit cell in the metasurface by regulating the on/off states of the switching components integrated in each unit cell. When illuminated by a monochromatic wave at frequency $f_{0}$, the metasurface reflects the incident wave to generate reflected signals that carry the encoded information, and the superposition of these reflected signals propagates as the final transmitted signals for communication. (b) Spreading process for bit streams. The bit streams to be transmitted are multiplied by their corresponding orthogonal spreading codes, yielding the final encoded streams. (c) Block diagram of the receiving process, in which the received signal captured by a single receiving antenna is first acquired by an oscilloscope for signal recording and storage and then downconverted, undergoes pilot‐aided carrier phase synchronization and is finally despread to recover the original messages.

whose time‐domain waveforms are shown in Figure 2b in detail. Because the bit rate of the spreading codes is eight times that of the original bit streams to be transmitted, the bit rate of the encoded streams is thus eight times higher than that of the original ones.

To further explore the beam shaping and SDM capability of the metasurface, the encoded streams $m_{n}\left(t\right)$ are superimposed with a preset gradient phase for beam deflection. Therefore, the coding sequences for the *k*th column become $M_{k}\left(t\right)=m_{n}\left(t\right)e^{j2\pi \phi_{k}}$, where $\phi_{k}$ is the gradient phase for the *k*th column. Assuming the metasurface functions in a slow‐varying regime, where the modulation frequency is significantly lower than the carrier frequency of the incident wave, the time‐varying reflection coefficient of the metasurface is approximately equal to its applied coding sequence, namely, ${\Gamma_{k}\left(t\right)=M}_{k}\left(t\right)$. Under the given assumptions, when a monochromatic harmonic wave at $f_{0}$ impinges on the metasurface, it is scattered into a modulated wave, which can be approximated as

(2) $$\begin{array}{c} s_{\text{scatt}}\left(\theta_{r},t\right)\approx \sum_{k}\Gamma_{k}\left(t\right)e^{jkd\;\sin\;\theta_{r}}e^{j{2\pi f}_{0}t}. \end{array}$$

where $\theta_{r}$ denotes the scattering angle of the reflected wave relative to the metasurface's normal direction and *d* represents the center‐to‐center spacing between adjacent columns of unit cells. This scattered wave $s_{\text{scatt}}\left(\theta_{r},t\right)$ thus contains all the multistream data after the spectrum‐spreading process, with all streams sharing the same spectral, spatial, and temporal resources.

The receiver architecture is depicted in Figure 2c. At the receiver, a single receiving antenna is used to capture the superposition of all encoded streams, which is first acquired by an oscilloscope for signal observation and recording before being subsequently subjected to downconversion and carrier phase synchronization. By leveraging prior knowledge of the orthogonal codes, the receiver can despread the signal one by one and recover the original transmitted message. The orthogonality of the codes ensures that each stream can be separated without interference from the others. In practice, this scheme supports two primary scenarios. First, multiple data streams can be intended for a single user, who can then receive all streams simultaneously. Alternatively, the streams can be directed to different users at various locations; in this case, each user can independently receive their designated data stream without interference from the others' streams. Notably, although 8‐bit Walsh codes are used in this work for proof‐of‐concept experimental verification, longer spreading codes can be readily introduced to accommodate a larger number of concurrent users.

### 2.2 Hybrid Multistream CDM and SDM Transmission Based on STCM

To elucidate the operating principle of the proposed STCM‐based wireless CDM, three different scenarios are studied, as illustrated in Figure 3. Figure 3 presents the encoded streams, coding matrices, and corresponding reflection patterns for three distinct communication scenarios. The first scenario considers multistream transmission to a single user. As depicted in Figure 3a, two independent data streams are encoded using orthogonal spread‐spectrum codes. To accommodate the two streams, the metasurface is partitioned into two independent regions, with Columns 1–4 forming Region 1 and Columns 5–8 forming Region 2 (Figure 3b). Each region is independently controlled by a binary stream, which is mapped to the metasurface phase via binary phase‐shift keying (BPSK). For a target receiving angle of $\theta_{r}=0{}^{\circ}$, no intercolumn phase gradient is required within each region. Consequently, both regions generate reflection patterns directed normally, as shown in Figure 3c. The user, equipped with a single antenna positioned at $\theta_{r}=0{}^{\circ}$, captures the superimposed signal and subsequently recovers the original streams via correlation despreading using the corresponding orthogonal codes. Notably, Supporting Information S1 proposes a simplified implementation alternative. Unlike conventional approaches requiring dedicated physical partitions for each data stream, this advanced design enables the entire metasurface to collectively encode superimposed data streams through joint amplitude‐phase modulation.

**FIGURE 3:**
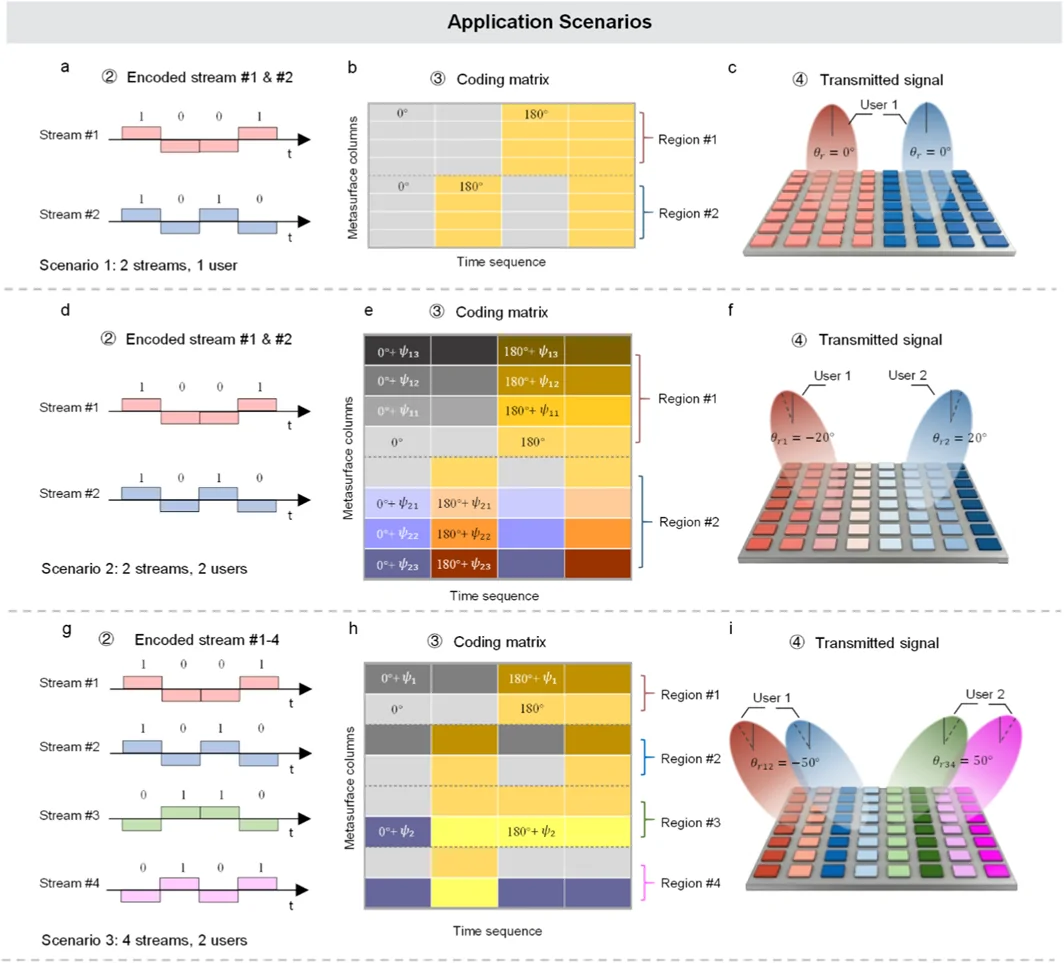
Encoded streams, coding matrices, and metasurface reflection patterns for three scenarios. (a) Encoded streams for Scenario 1. (b) Coding matrix for Scenario 1. The metasurface is divided into two regions, each modulated by one stream. Different colors indicate different reflection phases. (c) Radiation patterns of each region in Scenario 1, both pointing to 0°. (d) Encoded streams for Scenario 2. (e) Coding matrix for Scenario 2. The metasurface is divided into two regions, each modulated by one stream with an additional beam‐steering phase incorporated within the region. (f) Radiation patterns of Scenario 2, pointing to − 20° and 20°, respectively. (g) Encoded streams for Scenario 3. (h) Coding matrix for Scenario 3. The metasurface is divided into four regions, each modulated by one stream with an additional beam‐steering phase incorporated within the region. The beam‐steering phase distributions in Regions 1 and 2 are consistent, as well as those in Regions 3 and 4. (i) Radiation patterns of each region in Scenario 3. Regions 1 and 2 point to −50°, whereas Regions 3 and 4 point to 50°.

Although this scheme reduces coding complexity, it imposes stricter requirements on the amplitude‐phase regulation accuracy of individual unit cells.

The second scenario addresses the case where multiple users, located at distinct spatial positions, each receive a single data stream. Using the same two‐stream configuration (Figure 3d, identical to Figure 3a), the key distinction lies in the metasurface coding matrix design. As illustrated in Figure 3e, a beam‐steering phase $\psi_{ij}$ is applied to the *j*th column of the metasurface for the *i*th region, where *i* denotes the region index and *j* denotes the column index within the region. The detailed calculation of this beam‐steering phase is provided in Supporting Information S1. The reflection phase of each column is obtained by summing the beam‐steering phase and the BPSK‐modulated phase of the corresponding data stream, thereby deflecting each region's reflection pattern toward its target angle. Owing to the 2‐bit phase modulation constraint, the steering phases for the two regions are set to $\psi_{13}=\psi_{12}=90{}^{\circ}$, $\psi_{23}=\psi_{22}=90{}^{\circ}$, and $\psi_{11}=\psi_{21}=0{}^{\circ}$, with an intercolumn spacing of 18.5 mm. This configuration yields a reflection pattern for Region 1 directed at $\theta_{r1}=-20{}^{\circ}$ and for Region 2 at $\theta_{r2}=20{}^{\circ}$, as verified in Figure 3f. When a receiver is positioned at the target angle, the undesired signal from the other region is suppressed to a negligible level, ensuring that only the intended signal is effectively received. Subsequent despreading then recovers the original single data stream. Supporting Information S1 further expands this concept by introducing flexible partition designs tailored to target reflection patterns, enabling adaptation to complex spatial deployment scenarios.

The most comprehensive scenario involves multiple users, each located at a distinct spatial angle and requiring simultaneous reception of multiple streams. This configuration effectively combines the features of the two previous scenarios. Consider a case with four data streams, as shown in Figure 3g. Streams 1 and 2 are allocated to the target angle $\theta_{r12}$, whereas Streams 3 and 4 are allocated to $\theta_{r34}$. The metasurface is divided into four independent regions, each modulated by one data stream (Figure 3h). A critical design principle is that the regions serving the same receiver share an identical intercolumn phase gradient. Specifically, the two regions carrying Streams 1 and 2 are configured with a beam‐steering phase $\psi_{1}$, whereas those carrying Streams 3 and 4 use $\psi_{2}$. This ensures that reflection patterns for regions targeting the same angle are perfectly aligned, whereas those for different angles are steered toward distinct directions. For example, when both $\psi_{1}$ and $\psi_{2}$ are 90°, Regions 1 and 2 point to $\theta_{r12}=-50{}^{\circ}$, and Regions 3 and 4 point to $\theta_{r34}=50{}^{\circ}$. Figure 3i confirms that the patterns of regions corresponding to the same receiver completely overlap. A receiver deployed at the target angle thus captures the superimposed signal of its two assigned streams and subsequently separates them via orthogonal spread‐spectrum code despreading. However, such a spatial partitioning scheme inherently introduces an aperture‐scaling penalty. Quantitatively, equally dividing the *M*‐column metasurface into *N* subregions scales down the effective radiating aperture for each stream to 1/*N*. This reduction not only drops the directive gain but also, more crucially, intensifies inter‐region electromagnetic crosstalk as *N* increases, fundamentally degrading the code‐domain orthogonality. To mathematically validate the system feasibility under these dynamic hardware nonidealities, rigorous analytical models addressing the aperture‐scaling constraints and spreading code optimization are comprehensively provided in Supporting Information S1.

In summary, STCM‐based multistream transmission achieves crosstalk‐free communication in the code domain by leveraging both code orthogonality and spatial directivity. The introduction of beam‐steering phases enables seamless progression from single‐angle to multiangle multistream scenarios, offering a flexible and scalable framework for advanced wireless CDM systems.

### 2.3 Experimental Demonstration

To validate the proposed concepts and methodologies, a wireless communication testbed operating at 5 GHz is established in this study. The metasurface used is a 2‐bit programmable metasurface. From a system design perspective, 1‐bit phase resolution is theoretically sufficient to satisfy the basic requirements of both baseband modulation for CDM and beam steering for SDM. However, 2‐bit quantization offers irreplaceable advantages in practical implementations. Compared with the 1‐bit counterpart, the 2‐bit scheme not only provides additional degrees of freedom to support more precise beam steering but also accommodates multilevel digital modulation formats such as QPSK, thereby reserving room for further system performance enhancement.

As shown in Figure 4a, each unit cell has a side length of 18.5 mm and comprises two substrates and three metal layers. Three PIN diodes are soldered on the bottom layer of the unit cell. Specifically, simultaneous switching of PIN 1 and PIN 2 introduces a 90° phase shift in reflection between their on and off states, whereas an additional 180° phase shift is achieved through the switching of PIN 3. Figure 4b presents the simulated reflection phase under different biasing states of PIN diodes, denoted as 00, 01, 10, and 11, clearly demonstrating 2‐bit phase quantization characteristics. This phase information is then translated into the specific switching configurations of the three integrated PIN diodes via the mapping table (Table 2), which ultimately determines the high/low logic levels at the corresponding FPGA I/O ports—a high level turns a diode on, whereas a low level turns it off. Detailed information regarding the unit cell design and its working principle is found in Supporting Information S1 and also Ref. [46].

**FIGURE 4:**
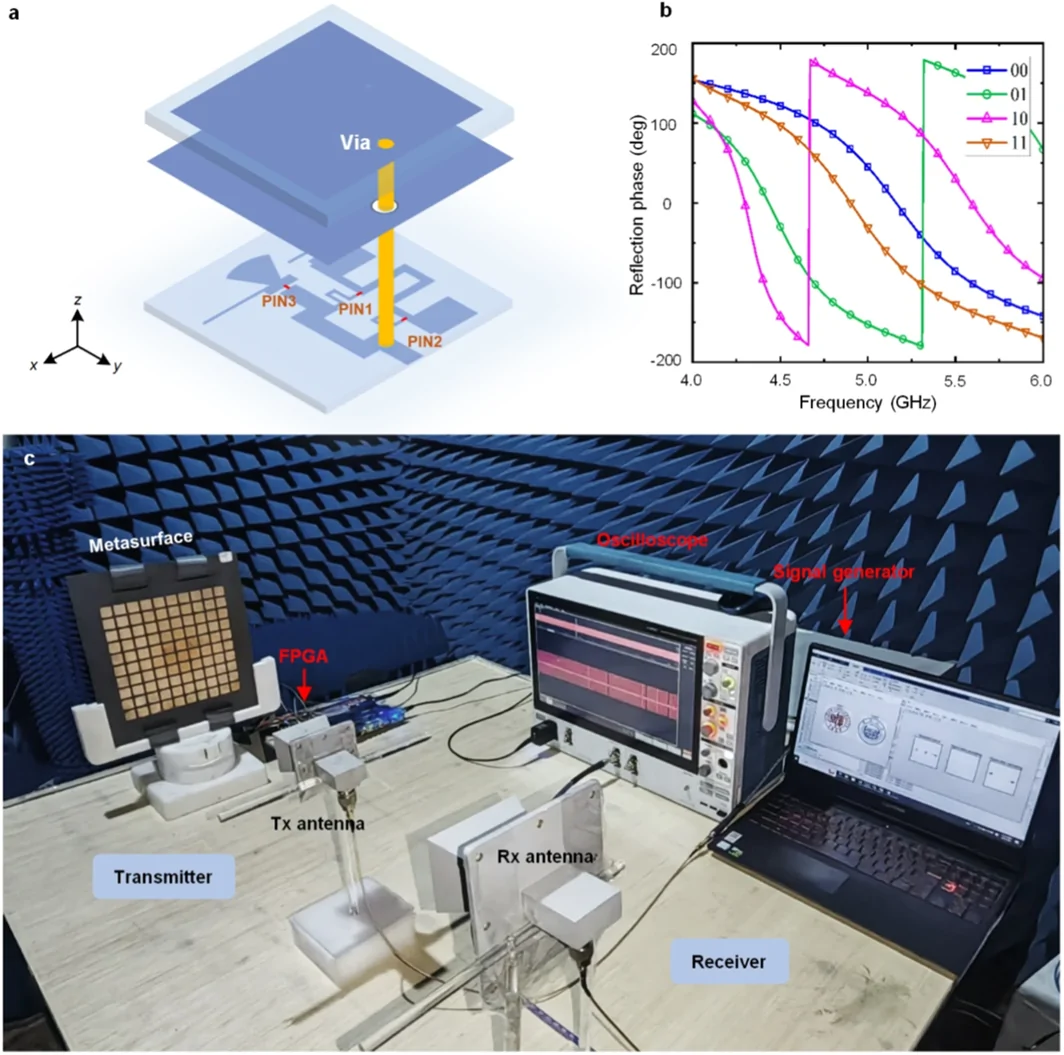
Details of the designed metasurface and experimental configuration. (a) Illustration of the space‐time coding metasurface unit cell. (b) Simulated reflection phase response of the unit cell. (c) Experimental configuration of the wireless communication testbed, capable of simultaneously transforming multistream data to various users. The CDM system operates at a chip rate of 5 MHz under a carrier frequency of 5 GHz.

**TABLE 2:** Correspondence between reflection phase, unit state, and PIN diode switching configuration.

Phase (deg)	States	PIN diodes	
		PIN 3	PIN 1/PIN 2
0	00	Off	Off
90	01		On
180	10	On	Off
270	11		On

Figure 4c shows the experimental configuration. The experiments are conducted in a microwave anechoic chamber to minimize unwanted electromagnetic reflections and external interferences. The transmitter comprises a signal generator (Agilent Technologies E8257D), an FPGA (Altera DE2‐115), a horn antenna, and a 10 × 10 metasurface array, where the metasurface dynamically reconfigures its properties through FPGA‐controlled real‐time coding sequence loading. The transmit horn antenna is aligned to the normal direction of the metasurface array and positioned 0.6 m from the metasurface. This distance is greater than the far‐field distance of the transmit horn to ensure that the metasurface is uniformly illuminated by the incident wave. The receiver uses a horn antenna positioned 1 m from the metasurface, with its angle relative to the metasurface adjustable as needed. The captured signals are acquired by a high‐speed oscilloscope (Tektronix MSO64B) and subsequently transferred via an Ethernet interface to a computer platform for postprocessing.

Experimental results of the multistream single‐user scenario are presented in Figure 5. In Scenario 1, the transmission of two streams is experimentally demonstrated by dividing the metasurface into left and right regions, each consisting of five columns. Each region is modulated independently using distinct signals, spread by orthogonal codes $c_{1}$ = [1,0,0,1,0,1,1,0] and $c_{2}$ = [1,0,1,0,0,1,0,1], respectively. Figure 5a presents the constellation diagram of the composite received signal, which results from the superposition of reflections from all unit cells. The spectrum of the transmitted signal exhibits clear spread‐spectrum characteristics: compared with the original prespread signal, the bandwidth of the received signal is expanded by a factor of eight, corresponding to the length of the spreading code (Figure 5b). Figure 5c,d presents the separated constellation diagrams and the corresponding decoded message from the two independent metasurface regions. All constellation points are clearly distinguishable, confirming the system's reliability for high‐quality image data transmission. Quantitatively, the measured postdespreading signal‐to‐interference‐plus‐noise ratio (SINR) extracted from these recovered constellations is 22.34 and 20.40 dB, showing excellent agreement with our physical link budget.

**FIGURE 5:**
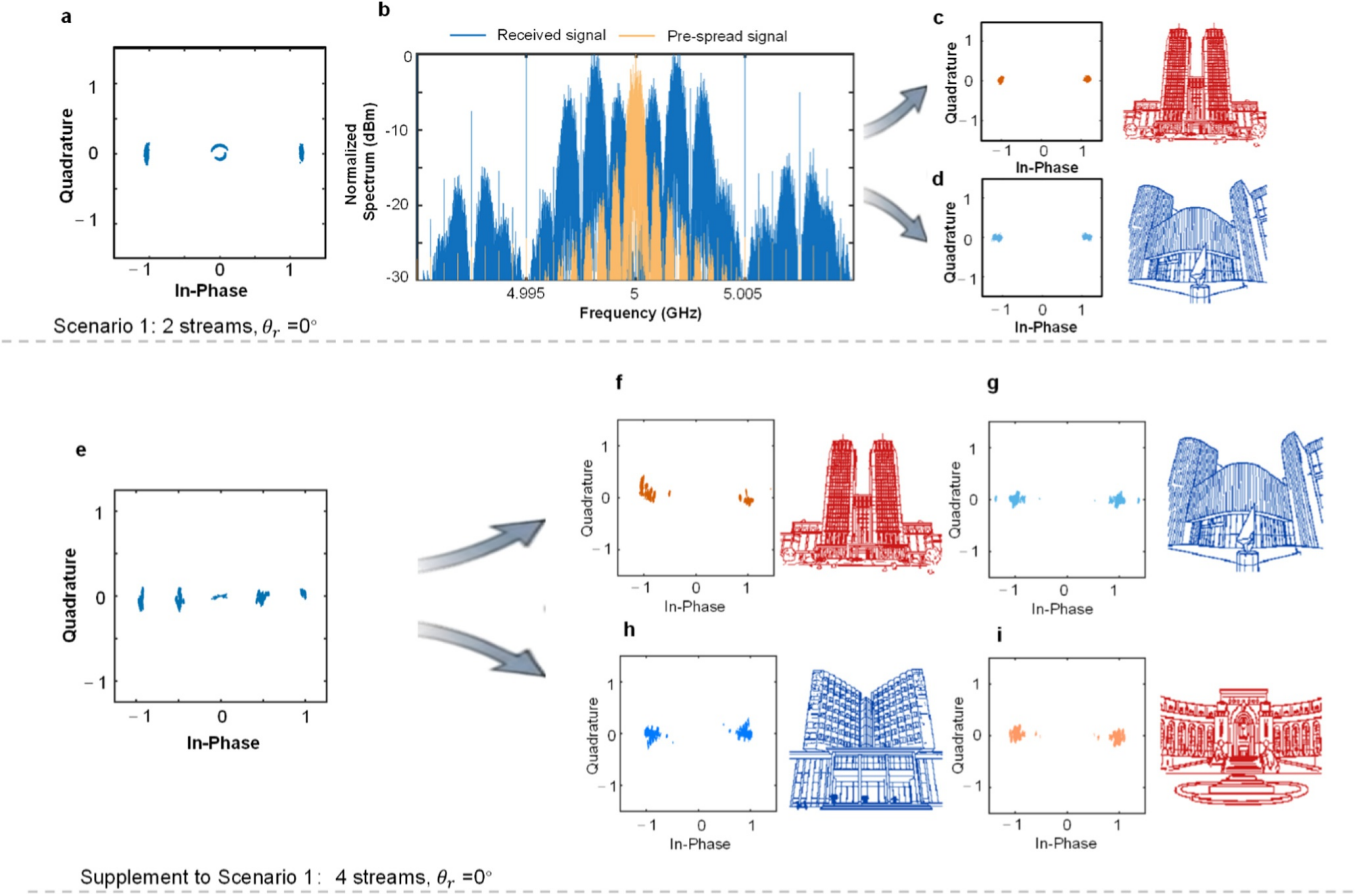
Measured constellations, frequency spectrum, and recovered images. (a) Scenario 1: measured constellation at the receiver. (b) Scenario 1: measured frequency spectra of the received signal and the prespread signal. (c) Scenario 1: constellation diagram of the despread data stream 1 and its recovered image. (d) Scenario 1: constellation diagram of the despread data stream 2 and its recovered image. (e) Supplement to Scenario 1: measured constellation at the receiver. (f) Supplement to Scenario 1: constellation diagram of the despread data stream 1 and its recovered image. (g) Supplement to Scenario 1: constellation diagram of the despread data stream 2 and its recovered image. (h) Supplement to Scenario 1: constellation diagram of the despread data stream 3 and its recovered image. (i) Supplement to Scenario 1: constellation diagram of the despread data stream 4 and its recovered image.

A four‐stream single‐user scenario is conducted as a supplement to Scenario 1; the metasurface is configured such that every two adjacent columns are modulated by the same transmitted information signal. The two remaining columns are covered with microwave‐absorbing material to eliminate unwanted reflections. The following orthogonal spreading codes are assigned to each stream:

$$c_{1}=\left[1,1,1,1\right]$$

$$c_{2}=\left[1,0,1,0\right]$$

$$c_{3}=\left[1,1,0,0\right]$$

$$c_{4}=\left[1,0,0,1\right]$$

Figure 5e shows the constellation diagram of the signal received by the horn antenna. Figure 5f–i presents the constellation diagrams and the recovered images corresponding to each independent region of the metasurface, with an SINR of approximately 18 dB. Although slight dispersion is observable in the constellations, the structures remain clearly distinguishable, confirming successful signal separation.

For the scenario where multiple users, located at distinct spatial positions, each receive a single data stream, the metasurface is partitioned as illustrated in Figure 3e. The two remaining columns are covered with microwave‐absorbing material to eliminate unwanted reflections. Figure 6a,c shows the constellation diagrams received by users at −20° and 20°, respectively. The observed clustering or dispersion in these constellations is attributed to the sidelobes in the reflection patterns of each region, where residual energy from undesired signals is not fully suppressed, thereby introducing interference to the intended signal. Figure 6b,d presents the constellation diagrams and the corresponding recovered messages corresponding to each independent region of the metasurface. The measured SINRs are 24.55 and 21.77 dB. Although slight dispersion remains observable in the constellations, the clustering structure is still clearly distinguishable, confirming the successful separation of the signals.

**FIGURE 6:**
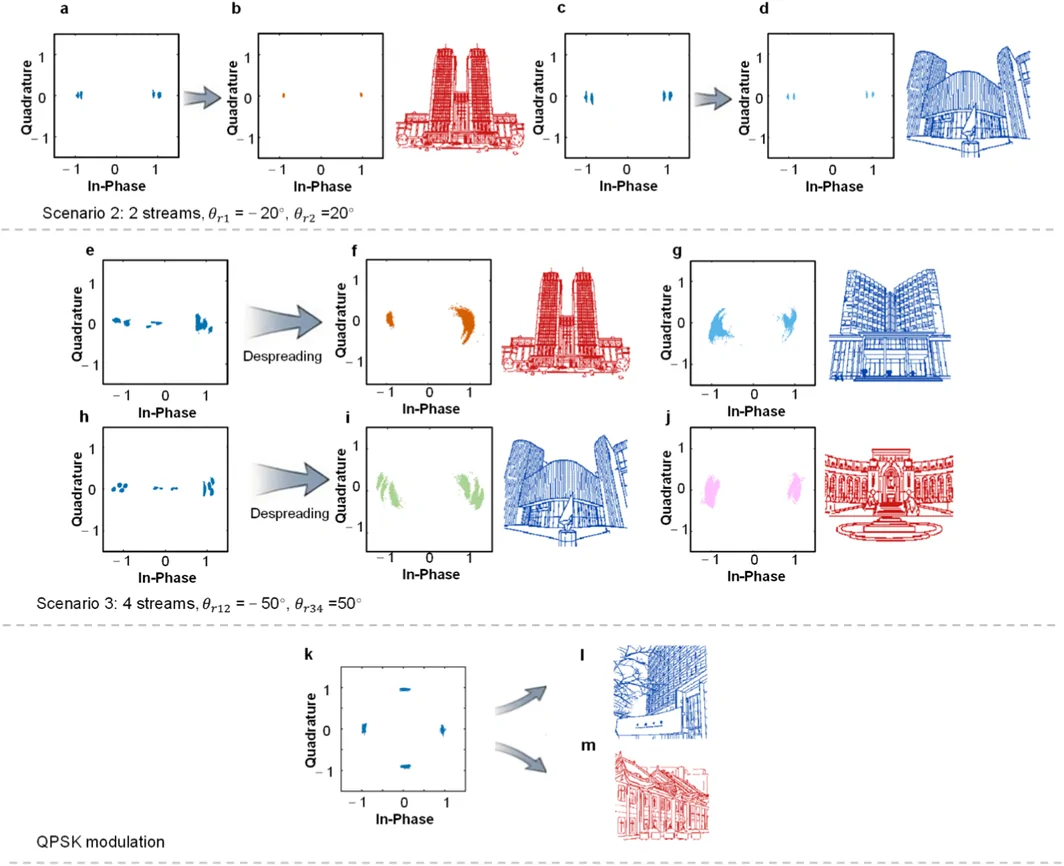
Measured constellations, frequency spectrum, and recovered images. (a) Scenario 2: measured constellation at $\theta_{r1}$. (b) Scenario 2: constellation diagram of the despread data stream 1 and its recovered image. (c) Scenario 2: measured constellation at $\theta_{r2}$. (d) Scenario 2: constellation diagram of the despread data stream 2 and its recovered image. (e) Case 3: measured constellation at $\theta_{r12}$. (f) Scenario 3: constellation diagram of the despread data stream 1 and its recovered image. (g) Case 3: constellation diagram of the despread data stream 2 and its recovered image. (h) Scenario 3: measured constellation at $\theta_{r34}$. (i) Scenario 3: constellation diagram of the despread data stream 3 and its recovered image. (j) Scenario 3: constellation diagram of the despread data stream 4 and its recovered image. (k) Measured constellation diagram at 0° of the received signal when QPSK is used. (l) Recovered images corresponding to stream 1 when QPSK is used. (m) Recovered images corresponding to stream 2 when QPSK is used.

For the four‐stream two‐user scenario where each user receives two data streams, the following orthogonal spreading codes are assigned to each stream:

$$c_{1}=\left[1,0,0,1,0,1,1,0\right]$$

$$c_{2}=\left[1,0,1,0,0,1,0,1\right]$$

$$c_{3}=\left[1,0,0,1,1,0,0,1\right]$$

$$c_{4}=\left[1,0,1,0,1,0,1,0\right]$$

The metasurface is partitioned as depicted in Figure 3e. Figure 6e,h presents the constellation diagrams received by users located at −50° and 50°, respectively. As the number of signals to be transmitted increases, the directivity of the radiation patterns diminishes because of the decreased number of columns in each region, resulting in increased dispersion in the received constellation diagrams. Nevertheless, this increase does not affect the correct discrimination of the signals. Figure 6f,g shows the demodulated constellation diagram and the corresponding original transmitted image for the signal received at −50°, whereas Figure 6i,j presents those for the signal received at 50°. The measured SINRs of the despread streams for the four‐stream scenario average approximately 17 dB. Benefiting from this robust postprocessing signal quality, the original messages can still be decoded flawlessly. In both cases, the images are accurately reconstructed. These experimental results confirm that spatially separated receivers can successfully and independently recover their intended data streams.

Furthermore, benefiting from the sufficient degrees of freedom offered by the 2‐bit phase‐quantized control, the established STCM platform natively scales to support QPSK transmission. In the QPSK experiment, the spreading codes are

$$c_{1}=\left[1,1,1,1\right]$$

$$c_{2}=\left[1,0,1,0\right]$$

The combined spread‐spectrum signal would exhibit four possible states over one spreading code period, corresponding to the data pairs (00, 01, 10, 11), where the first and second digits represent the spread data bit in Stream 1 and Stream 2, respectively. Four reflection phase states of a 2‐bit metasurface unit are designed to correspond exactly to these four information combinations, which can also be regarded as QPSK modulation.

The test results are demonstrated in Figure 6k–m. The measured constellation diagram at receiving angle θ=0° exhibits four clearly distinguishable clusters. The SINR of the received signal is 19.88 dB. Figure 6l,m presents the successfully recovered original data and the corresponding reconstructed images. Both the constellation diagrams and image recovery results confirm the feasibility of integrating metasurface technology with CDMA for multiuser signal modulation, enabling the recovery of all user information through a single receiver channel.

## 3 Discussion

This study presents a hybrid code‐division wireless communication system based on an STCM, capable of simultaneously transmitting multistream messages to the same receiver while enabling SDM. Unlike previous CDM systems that rely on intricate RF chains or metasurface‐enabled multiplexing systems, the proposed scheme achieves secure multiuser transmission with enhanced spectral efficiency using simplified, low‐cost RF front‐ends. To demonstrate that multistream signals received by a single channel can be reliably recovered through code‐domain separation, experimental validation confirms the successful transmission of single or dual images to a single user or two distinct users at a prespecified angle. The presented work establishes a new paradigm for applications in scenarios requiring low‐power, high‐density access.

## 4 Materials and Methods

### 4.1 Generation, Reception, and Processing of Data Streams

To generate bit streams from image data, each pixel is mapped to one bit of information: colored pixels are encoded as 1 and white pixels as 0. To acquire the generated bit stream, the oscilloscope's sampling rate is set to 1.5625 GS/s, which is equivalent to downconverting the received signal to 312.5 MHz. Further downconversion is conducted using MATLAB. During transmission, a 2000‐bit frame header with a bit rate of 5 MHz is adopted, consisting of 1000 successive ‘0’s followed by 1000 successive ‘1’s. When processing the received data using MATLAB, the phase of the successive 1s in the frame header is taken as the reference phase, and the phase of the received signal is compared with this reference phase to achieve phase alignment.

## Author Contributions

**Yuzhen Chen:** conceptualization, methodology, investigation, writing – original draft, writing – review and editing, validation, formal analysis, data curation. **Xiaoyi Wang:** writing – review and editing, conceptualization, methodology. **Guo‐Min Yang:** supervision, project administration, resources, funding acquisition. **Ya‐Qiu Jin:** supervision, resources.

## Funding

The authors acknowledge financial support from the National Natural Science Foundation of China under Grant 62471146 and the State Key Laboratory of Radio Frequency Heterogeneous Integration Open Scientific Research Program No. KF2024017.

## Conflicts of Interest

The authors declare no conflicts of interest.

## Supporting information


Supporting Information S1

## Data Availability

The data that support the findings of this study are available from the corresponding author upon reasonable request.
