# Supplementary material for: Radio‐Frequency Front‐End‐Free Hybrid Code‐Division and Space‐Division Wireless Multiplexing Using Space‐Time‐Coding Metasurface
Source: Nanophotonics. 2026 Aug 31;15(17):e70277. doi: 10.1002/nap2.70277 (PMC13672144; doi:10.1002/nap2.70277)
Supplement: Supplementary file 1 [file NAP2-15-e70277-s001.docx]

Supplementary Information for

**Radio-Frequency Front-End-Free Hybrid Code-Division and Space-Division Wireless Multiplexing Using Space-Time-Coding Metasurface**

**This supplementary information includes:**

Supplementary Note 1. Amplitude-phase tunable metasurface for CDM communication

Supplementary Note 2. Calculation of the beam steering phase

Supplementary Note 3. Extended partition schemes for the STCM

Supplementary Note 4. Theoretical analytical model of SINR

Supplementary Note 5. Quantitative analysis of spreading code length extension on SINR performance

Supplementary Note 6. Detailed analysis of the metasurface unit cell

**Supplementary Note 1.** **Amplitude-phase tunable metasurface for CDM communication**

Fig. S1. (a) Schematic of the metasurface measurement setup. (b) Received constellation diagram obtained when the receiving horn antenna is positioned at 0°. (c) Recovered images corresponding to stream 1. (d) Recovered images corresponding to stream 2.

Table S1. State Configurations metasurface for Partition-Free Two-Stream Superposition Transmission

| Stream 1 | Stream 2 | Reflection Amplitude | Reflection Phase |
| --- | --- | --- | --- |
| 0 | 0 | high | 0° |
| 0 | 1 | low | - |
| 1 | 0 | low | - |
| 1 | 1 | high | 180° |

For implementing CDM communication, one feasible approach is to partition the metasurface into subarrays. However, increased partitioning introduces challenges in maintaining uniform reflection amplitude across all regions and consistent unit performance within each region, primarily attributed to fabrication tolerances. A more straightforward alternative is to enable metasurface unit cells to directly represent the composite state of multiple superimposed encoded streams.

Herein, we employ a metasurface with independently tunable 2-bit reflection and 1-bit transmission states. Since the reflection amplitude can be regarded as 0 when the unit operates in the transmission state, this metasurface can be equivalent to an amplitude-phase tunable metasurface with 1-bit amplitude tunability and 2-bit phase tunability in reflection mode. As illustrated in Table S1 this metasurface enables the transmission of the superposition of two encoded streams without the need for partitioning.

Fig. S1a illustrates the measurement setup of the metasurface, while Fig. S1b presents the received constellation diagram when the receiving horn antenna is positioned at 0°. Figs. S1c and S1d correspond to the recovered images of stream 1 and stream 2, respectively. The intact recovery of the images verifies the feasibility of the proposed communication system.

**Supplementary Note 2. Implementation of the beam steering phase**

When the STCM is illuminated by a normally incident plane wave, the phase distribution required for each region’s pattern points towards ${\hat{u}_{0}(\theta}_{r},\varphi_{r})$ is given by:

$$\begin{aligned} \psi_{m,n}=-k_{0}\vec{r}_{mn}\cdot\hat{u}_{0}+\psi_{0}\#\text{(}\text{S}\text{1)} \end{aligned}$$

where $\vec{r}_{mn}$ is the position vector of the *mn*th element and $\psi_{0}$ is an optimized initial phase for beamforming performance. The calculated phase$\psi_{m,n}$ is subsequently wrapped into the range of 0° to 360°. With the 2-bit phase quantization implemented, the quantization phase is defined as

$$\begin{aligned} \psi_{m,n}^{2-bit}=\left\{ \begin{aligned} \begin{matrix} 0^{\circ}, 0^{\circ}\leq\psi_{m,n}<45^{\circ} or 315^{\circ}\leq\psi_{m,n}<360^{\circ} \\ 90^{\circ}, 45^{\circ}\leq\psi_{m,n}<135^{\circ} \end{matrix} \\ \begin{matrix} 180^{\circ}, 135^{\circ}\leq\psi_{m,n}<225^{\circ} \\ 270^{\circ}, 225^{\circ}\leq\psi_{m,n}<315^{\circ} \end{matrix} \end{aligned} \right.\#\text{(}\text{S}\text{2)} \end{aligned}$$

Table S2. Steering phase STCM Reflected Patterns to $\theta_{r}$=0°–50°

| $\theta_{r}$($^{\circ}$) | Beam steering phase ($^{\circ}$) |
| --- | --- |
| 0 | $0^{\circ}$,$0^{\circ}$,$0^{\circ}$,$0^{\circ}$ |
| 10 | $0^{\circ}$,$0^{\circ}$,$0^{\circ}$,$90^{\circ}$ |
| 20 | $0^{\circ}$,$0^{\circ}$,$90^{\circ}$,$90^{\circ}$ |
| 30 | $0^{\circ}$,$90^{\circ}$,$90^{\circ}$,$180^{\circ}$ |
| 40 | $0^{\circ}$,$90^{\circ}$,$180^{\circ}$,$180^{\circ}$ |
| 50 | $0^{\circ}$,$90^{\circ}$,$180^{\circ}$,$270^{\circ}$ |

Fig. S2. Calculated reflected patterns at 5 GHz of the STCM region, corresponding to Table S2.

When a region comprises four columns with an inter-column spacing of 18.5 mm, the steering phase calculated to steer the reflected pattern to deflection angles of $\varphi_{r}$=0° and $\theta_{r}$ ranging from 0° to 50° are presented in Table S2. The corresponding radiation patterns are illustrated in Fig. S2. In constructing the coding matrix, steering the beam to the desired direction can be achieved simply by superimposing the steering phase onto the phase associated with the encoded stream.

**Supplementary Note 3. Extended partition schemes for the STCM**

Fig. S3. Calculated reflection patterns at 5 GHz when (a) region 1 (the blue region) transmits encoded stream 1 and (b) region 2 (the yellow region) transmits encoded stream 2. The main lobes of the total pattern remain directed at 0° and ±54.2°.

Fig. S4. Schematic of the SDM-CDM communication system. This figure illustrates the directional modulation capability of the metasurface in this operating mode, where only the target user in the main beam direction can successfully decode the information using the corresponding spread spectrum codes, while eavesdroppers at other locations experience complete information loss.

In addition to the previously mentioned approach of treating adjacent columns as a single region, metasurfaces can also adopt more diverse partitioning forms to achieve the desired pattern. Figure S3 illustrates a feasible partitioning scheme: the 1st, 3rd, 5th, 7th, and 9th columns (blue regions) of the metasurface are modulated by encoded stream 1, while the 2nd, 4th, 6th, 8th, and 10th columns (yellow regions) are modulated by encoded stream 2. According to the generalized Snell’s law, steering phases are introduced separately within the blue and yellow regions to control the angles of the scattered beams.

As a demonstration, Fig. S3a presents the reflection pattern when the blue regions are activated and exhibit a 0° phase difference between adjacent columns. The spacing between adjacent blue regions is twice the unit width (37 mm), and the pattern’s main lobe is directed at *θr* = 0° with a half-power beamwidth (HPBW) of 19°. This beamwidth is narrower than that when the units are contiguous (i.e., with a spacing of 18.5 mm), indicating better directivity. Fig. S3b presents the reflection pattern when the yellow regions are activated and maintain a 180° phase difference between adjacent columns. This configuration results in main lobes oriented at *θr* = ±54.2° and a HPBW of 30°.

To demonstrate the feasibility of the proposed partitioning scheme, a dual-user SDM experiment is performed. Specifically, Stream 1 is transmitted to 0°, while stream 2 is transmitted to 55°. The received waveforms at 0°, 30°, and 55° are measured, and the constellation diagrams are plotted while recovering the transmitted images. As shown in Fig. S4, legitimate users at 0° and 55° exhibit well-defined constellation patterns, successfully reconstructing the images corresponding to stream 1 and stream 2, respectively. In contrast, the eavesdropper at 30° received a scattered constellation diagram without clustering in the two 180° phase-separated regions, rendering information decoding impossible.

**Supplementary Note 4.** **Theoretical analytical model of SINR**

To mathematically verify the feasibility of the proposed system under physical sub-aperture geometric non-idealities and code-domain non-orthogonal characteristics, this section constructs a rigorous Signal-to-Interference-plus-Noise Ratio (SINR) theoretical model.

When a metasurface with a total of *M* columns is physically and equally partitioned into *N* sub-apertures to support *N* concurrent data streams, the received signal power of each stream is governed by two physical mechanisms: aperture power splitting and directive gain degradation. Specifically, partitioning the aperture reduces the geometric area of each sub-aperture, meaning that the feed-illuminated power captured by each sub-aperture scales down to 1/*N*. Concurrently, the reduced number of columns per sub-aperture broadens the radiation beamwidth, leading to a drop in gain. More importantly, due to the inherent hardware constraints of the 2-bit meta-atoms, the programmable reflection states inevitably deviate from the ideal equal-amplitude and exact $90^{\circ}$ phase-shift profile. Such phase quantization and amplitude distortions fundamentally impair the physical orthogonality of the spreading sequences during the space-time reflection process. Consequently, the self-correlation of the intended data stream degrades, introducing a self-correlation scaling factor $\rho_{i,i}$ that attenuates the desired signal power. Meanwhile, the cross-correlation between unintended data streams and the target code fails to vanish to zero, thereby introducing a residual cross-correlation error $\rho_{m,i}$. As the number of sub-apertures *N* increases, the inter-region electromagnetic crosstalk and code-domain mutual couplings are heavily aggravated, which further worsens the self-correlation degradation $\rho_{i,i}$ and amplifies the non-orthogonal interference error $\rho_{m,i}$.

Taking into account these effects, the exact SINR expression for the *i*-th co-axial data stream is derived as follows:

$$\begin{aligned} \mathrm{SINR}_{i}=\frac{\left| \rho_{i,i} \right|^{2}\frac{1}{N}P_{rx,0}\left| F_{i}\left( \theta_{i} \right) \right|^{2}}{\frac{N_{0}B}{L}+PL\cdot\left[ \left( 1-\left| \rho_{i,i} \right|^{2} \right)\frac{1}{N}P_{rx,0}\left| F_{i}\left( \theta_{i} \right) \right|^{2}+\sum_{m\neq i}^{N} \left| \rho_{m,i} \right|^{2}\frac{1}{N}P_{rx,0}\cdot\left| F_{m}\left( \theta_{i} \right) \right|^{2} \right]}\#\text{(S3)} \end{aligned}$$

Where:

- *L*: The sequence length of the bipolar pseudo-random spreading codes.
- *B*: The total physical bandwidth of the space-time coded signal modulation.
- $N_{0}$: The single-sided noise power spectral density at the receiver front-end, which is primarily determined by the thermal noise floor of the receiver hardware.
- $P_{rx,0}$: The nominal reference peak received power when the full aperture is unpartitioned and transmits a single stream.
- $F_{i}(\theta_{i})$: The gain of the radiation pattern of the *i*-th target sub-aperture at the target demodulation angle$\theta_{i}$.
- $F_{m}(\theta_{i})$：The level of the gain of the *m*-th adjacent interfering sub-aperture at the target user angle $\theta_{i}$.
- $\rho_{i,i}$: Represents the hardware complex-amplitude distortion efficiency of the *i*-th target stream, which decreases with the increment of partition number *N*.
- $\rho_{m,i}$: Represents the mean-square value of the residual cross-correlation between the *m*-th unintended stream and the *i*-th target code under partition state *N*. Crucially, $\rho_{m,i}$ scales up as *N* increases, because more sub-aperture partitions aggravate inter-region electromagnetic crosstalk

Fig. S5. Single-user equivalent SINR evolution versus spatial channel path loss across different sub-aperture partitioning configurations (N = 1, 2, 10).

Fig. S5 illustrates the evaluation of the single-user equivalent SINR as a function of the spatial channel path loss under different sub-aperture partitioning configurations (N = 1, 2, 10). The path loss axis is reversed to intuitively represent the link progression from a distant/weak-signal regime (left, 120 dB) to a near-field/strong-signal regime (right, 70 dB). All data streams across different sub-apertures are transmitted toward $\theta=0^{\circ}$.

The results demonstrate two primary trends. First, at a minimum path loss of 70 dB, the single-user SINR decreases monotonically as the number of concurrent data streams increases. Specifically, the peak SINR drops from 29.99 dB under single-stream transmission (N=1) to 21.92 dB under dual-stream concurrency (N=2), and eventually degrades to 11.87 dB when the network scales up to ten concurrent streams (N=10). Second, as the wireless link extends into the distant weak-signal region, the single-user SINR across all configurations exhibits a sharp, linear decline with the increase of channel path loss.

In experienments, severe inter-column mutual coupling and hardware crosstalk inevitably degrade the orthogonality between individual data streams at high concurrency levels in experiments, causing the measured SINR to fall short of these ideal theoretical upper bounds. Consequently, the hardware experimental verification is tightly capped at a maximum of four concurrent streams.

Fig. S6. Equivalent SINR of the target user (fixed at $\theta_{1}=0^{\circ}$) as a function of the adjacent interference beam steering angle $\theta_{2}$ under a dual-stream configuration *N*=2.

Fig. S6 valuates the impact of spatial beam steering separation on the single-user equivalent SINR under a constant user count profile N=2. While the target user’s data stream remains tightly focused along the boresight axis ($\theta_{1}=0^{\circ}$), the steering angle of the adjacent interfering data stream $\theta_{2}$ is continuously scanned from $0^{\circ}$ to $90^{\circ}$.

A prominent trend observed is the swift initial rise in the target user’s SINR as the interference beam angle $\theta_{2}$ deviates from $0^{\circ}$. At $\theta_{2}=0^{\circ}$, the target user experiences direct mainlobe jamming, yielding the minimum SINR threshold. As $\theta_{2}$ increases, the target user rapidly exits the intense mainlobe profile of the interfering channel, causing a subsequent escalation in the demodulated SINR.

However, as the interference angle expands further into wider angular ranges, the SINR does not maintain a monotonic increase but exhibits a slight localized degradation and a ripple-like behavior. This fluctuation is inherently governed by the spatial sidelobe scanning effect of the discrete sub-aperture array. As the interference mainbeam steers farther away, its peripheral radiation sidelobes (specifically the first-order minor lobe) inevitably sweep across the $0^{\circ}$ boresight axis, injecting transient residual leakage back into the target user’s channel and causing SINR recessions.

**Supplementary Note 5.** **Quantitative analysis of spreading code length extension on SINR Performance**

Fig. S7. Impact of spreading code length extension on single-user equivalent SINR versus channel path loss.

Fig. S7 uantitatively demonstrates that extending the spreading code length *L* yields a higher single-user equivalent SINR across all path loss regimes. Under a dual-stream configuration (*N*=2) where all concurrent data streams are steered toward the broadside, the single-user SINR at a minimum path loss of 70 dB elevates from 20.74 dB under the short code configuration (L=8) to 21.92 dB under the long code configuration (L=128). This SINR enhancement is fundamentally driven by the attenuation of thermal noise power $\frac{N_{0}B}{L}$ as well as the suppression of the residual cross-correlation floor $\left| \rho_{m,i} \right|^{2}$.

**Supplementary Note 6. Detailed analysis of the metasurface unit cell**

Fig. S8. The unit cell configuration of the metasurface. All diodes employed in the structure are MADP000907, with their equivalent circuit model presented in the bottom-left corner of the figure (*R*=5.2 Ω, *L*= 30 pH, *C*=25 fF).

Fig. S8 illustrates the unit cell structure of the metasurface. As depicted, the metasurface unit cell comprises two substrate and three metal layers, with the dielectric material being F4B (*εᵣ*= 2.55). The side length of the unit cell is 18.5 mm. At the top of the metasurface, a patch antenna serves as the electromagnetic wave receiver and radiator. This patch antenna is connected to a reflective phase shifter at the bottom of the unit cell by a via. The side length of the top metal patch is 15 mm.

Fig. S9. Bottom-layer structure of the metasurface unit cell

Table S3. The parameters of metasurface unit cell

| Parameter | *l*_1_ | *l*_2_ | *l*_3_ | *l*_4_ | *l_5_* | *l_6_* | *l_7_* | *w*_1_ | *w*_2_ | *r* |
| --- | --- | --- | --- | --- | --- | --- | --- | --- | --- | --- |
| Value (mm) | 4.35 | 7.2 | 4.35 | 2 | 12 | 4.2 | 3.8 | 1.6 | 3 | 4.2 |

Table S4. State correspondence between metasurface unit cells and diode ON/OFF configurations

| States | PIN Diodes | |
| --- | --- | --- |
|  | PIN 3 | PIN 1 / PIN 2 |
| 00 | OFF | OFF |
| 01 |  | ON |
| 10 | ON | OFF |
| 11 |  | ON |

Fig. S9 depicts the bottom-layer configuration of the proposed metasurface unit cell, functioning as a reflective phase shifter. Detailed parameters are presented in Table S3. Simultaneous switching of PIN1 and PIN2 introduces a 90° phase shift in reflection between their ON and OFF states. The sector-shaped stub connected to PIN3 acts as a short circuit at the operating frequency, enabling an additional 180° phase shift through PIN3 switching. Table S4 summarizes the operational state mappings of the metasurface unit cell corresponding to different diode switching configurations.

(a)	(b)

Fig. S10. Simulation results of the proposed 2-bit unit cell. a Reflection amplitude of 2-bit unit cell for *x*-polarized incidence and *y*-polarized incidence. **b** Reflection phase of 2-bit unit cell for *x*-polarized incidence and y-polarized incidence.

As illustrated in Fig. S10, the reflection phases of the metasurface unit cell is simulated under normal incidence in CST Microwave Studio 2023. It can be observed that the reflection loss is below 2 dB, and phase differences between adjacent states of the 2-bit metasurface unit cell are approximately 90° across various reception angles at 5 GHz.
